# Harnessing the antigenic fingerprint of each individual cancer for immunotherapy of human cancer: genomics shows a new way and its challenges

**DOI:** 10.1007/s00262-013-1422-x

**Published:** 2013-04-19

**Authors:** Pramod K. Srivastava, Fei Duan

**Affiliations:** Department of Immunology, Carole and Ray Neag Comprehensive Cancer Center, University of Connecticut School of Medicine, 263 Farmington Avenue, Farmington, CT 06030 USA

**Keywords:** Personalized medicine, Individualized medicine, Bio-informatics, Immunomics, CIMT2012

## Abstract

The idea that individual tumors are antigenically unique has been around since the very dawn of our recognition of adaptive immune response to tumors. That idea has inspired a small number of attempts at individualized immunotherapy of human cancers. Such previous attempts for solid tumors have been hobbled by an inability to define the individually unique antigenic repertoire of tumors because of technological difficulties. The new availability of rapid and cheap high throughput DNA sequencing promises to overcome that hurdle. Using this new ability, coupled with bio-informatic tools, it is now possible to define the immunogenic repertoire of any tumor to a high degree of granularity within a practical time frame and an acceptable cost. The development of these ideas, and a small number of such studies that underscore this promise, is discussed. This new way—of characterizing the tumor immunome through characterization of the tumor genome—has distinct challenges, including selection of the appropriate peptides, choosing methods of immunizations that can incorporate tens of epitopes, and addressing issues of antigenic heterogeneity of tumors. However, tools for meeting these challenges exist and are emergent.

## Tumors as seen by T lymphocytes

T cells are the prime movers of the endogenous immune response to cancer, although they may be aided (or hindered) in this process by other cells. Although a number of antibodies to molecules expressed on tumors (and normal cells) are now used to treat cancers, they are used as pharmacological rather than immunological agents. All the immunological agents approved for treatment of human cancers activate the T cell responses to cancers [1–3]. This discussion will therefore focus purely on the T cell epitopes of cancers and the responses elicited by them.

Much of our initial understanding about T cell epitopes came from study of viral immunity. T cell epitopes of viruses can be identified and can be used to elicit immune responses and protective immunity against viruses. As it became possible to generate T cells against mouse and human tumors, it was expected that identification of epitopes of cancers could be similarly used to elicit immune responses and protective immunity against cancers. It has now been over 20 years since it became possible to identify the T cell epitopes of mouse and human cancers of non-viral origins [4], and a large number of T cell epitopes have now been defined and characterized [5].

Such epitopes have fallen into two categories, one where the epitopes seen by the antitumor T cells are identical between normal and tumor cells, and second, where such epitopes are specific to the tumor cells and not seen in normal cells, by virtue of a tumor-associated mutation or other genetic event. The former class of the T cell tumor epitopes, the shared tumor epitopes, so-called because they are shared by tumors and by tumors and normal cells, has generated much of the enthusiasm and activity over the last 20 years. To be clear, the shared epitopes themselves consist of two sub-classes of epitopes—the differentiation antigens (such as tyrosinase), which are shared between normal tissues and tumors, and the “cancer testes” or CT antigens (such as MAGE and NY-ESO1), which although un-mutated are expressed on germinal tissues and cancers, but not on normal adult tissues. It is important to point this here because the CT antigens, although un-mutated, are tumor specific (if one discounts the germinal tissues). The shared epitopes have been tested extensively for their ability to immunize and generate T cell responses and to protect mice and men against cancers. Notwithstanding a lone voice or two [6–8], the fact that these shared epitopes are not tumor specific, and hence may not be immunogenic or immune protective, has been mostly glossed over. (It is useful to remember that the T cell epitopes of viruses were of course all virus specific.) Indeed, the evidence from mouse models lends credence to the idea that the lack of tumor specificity of these epitopes is a barrier to their ability to elicit immune-protective anti-tumor responses [9, 10]. Instead, the argument has been that since these epitopes are common between tumors and normal tissues, and since there must exist a degree of tolerance to the antigens, the goal should be to break tolerance against such self-antigens. The possibility that such breaking of tolerance, if achieved, would lead to unacceptable toxicities, has not been generally considered to be a major problem. Since the studies carried out thus far have failed to elicit potent antitumor responses, or potent autoimmunity for that matter, the issue of toxicities remains moot. However, two large randomized multi-center clinical trials, actually the largest ever trials in the history of cancer vaccination, are currently testing whether immune response to one such shared tumor antigen, MAGE, elicits clinical benefit in cancer patients. The outcome of these trials will reveal if immunization with the shared, non-tumor-specific epitopes is tumor protective; if the answer is in the affirmative, the results will also reveal, if such immunizations elicit pathological autoimmunity. Regardless of the outcome those results shall be instructive.

The latter class of T cell epitopes, the ones where the epitopes are tumor specific by virtue of the fact that a mutation in a normal sequence has created a new epitope, has been problematic as well: an overwhelming proportion of these mutations is found in only a given tumor, that is, the epitopes are individually distinct for a tumor. Although immunization with such tumor specific epitopes in mouse models of cancer has shown them to be highly tumor protective for the tumor that harbor them [11–15] (Table 1), and the indirect evidence in humans has been tantalizing [16], what does one do with an individually unique epitope even though it is tumor specific and perhaps even immune protective against a tumor? How does one generate a vaccine for just one tumor? The prospect of generating T cells from individual patients, characterizing the individually unique tumor-specific epitopes from these T cells for each patient, and immunizing each patient with such epitopes, is simply not practical for a variety of obvious reasons. For these good reasons, this latter class of epitopes has not elicited much enthusiasm.

**Table 1:** T-cell-defined epitopes of mouse tumors and their characteristics

Protein	Tumor(s)	MHC allele	Peptide sequence	Unique or shared	Elicits tumor rejection?	References
L9 ribosomal protein	6132A squamous carcinoma	IE^k^	DFNHINVELS*H*LGK	Unique	Yes	[11]
P68 helicase	8101 squamous carcinoma	K^b^	SNFV*F*AGI	Unique	Yes	[12]
P53	Meth A fibro-sarcoma	K^d^	KY*I*CNSSCM	Unique	Yes	[13]
ERK2	CMS5 fibro-sarcoma		L*Q*IHSANVL	Unique	Yes	[14]
L11 ribosomal protein	Meth A fibro-sarcoma	IE^d^	EYELRK*H*NFSDTG	Unique	Yes	[15]
P1A	Many	L^d^	LPYLGWLVF	Shared	No^a^	[9, 10]
AH1	Many	L^d^	SPSYVYHQF	Shared	No	[54], Un-published

The letter in italics denoted the altered residue created by a mis-sense mutation

^a^P1A has been shown to mediate tumor rejection if P1A and B7-1 expressing cells are used as vaccines [53]

To sum up the above, there are powerful scientific reasons and data against the idea that the shared tumor antigens may elicit protective tumor immunity; however, the denouement for this line of thinking is not far off: the two randomized trials with the shared MAGE antigen expect to be un-blinded within the next two years. The idea that the individually unique tumor antigens may be tumor protective is more appealing theoretically and is supported by considerable mouse data and some human evidence; however, it appears at first blush, to be logistically untenable. As a matter of fact, efforts to harness the individuality of immunogenicity of each cancer have a rather long and interesting history. This is discussed in the next section, followed by an overview of the extraordinary opportunities now available for this pursuit because of the availability of high throughput DNA sequencing technologies.

## Harnessing the individually distinct immunome of each individual cancer: some “medieval” and “modern” history

The first hint that T cells may be recognizing individually specific mutations in each individual cancer came long before we knew of T cells. Prehn and Main [17], Klein et al. [18] and others noted over 50 years ago that inbred mice could be immunized against syngeneic tumors, even autochthonous tumors, and that such immunity was individually specific: tumors of the same histological type, induced by the same carcinogen in mice of the same haplotype, still showed individually distinct antigenicity. In a dramatic demonstration, Globerson and Feldman [19] showed that two tumors induced on each flank of a single mouse by two independent injections of the same carcinogen were individually distinct antigenically. Basombrio [20] tested this individuality in a large panel of 25 tumors and observed the “extreme rarity of either totally or partially shared antigenic components between methylcholanthrene-induced tumors, as demonstrated by rejection of tumor cell inocula.”

These observations starting well over 50 years ago suggested that each time there was a new transforming event, there was a new and unique pattern of immunogenicity. One of the possibilities considered at the time was that this uniqueness of immunogenicity was simply a reflection of pre-existing unique patterns of immunogenicity in the normal cells. Clever experiments, which tested the patterns of immunogenicity of cells, transformed in vitro with the same carcinogen soon suggested otherwise: They showed that progeny of the same normal cell, transformed in vitro, had unique patterns of immunogenicity [21]. These ideas have simply stayed in the literature for lack of avenues to explore them, until now, as discussed in the next section.

And now for the “modern” history. A number of human studies have attempted to harness the individually distinct immunogenicity of individual human tumors. Three examples will illustrate the point. B cell lymphomas present a unique opportunity (no pun intended) because the tumors each have a unique idiotype (antigen). Building on the pioneering studies of Ronald Levy using anti-idiotypic antibodies to treat B cell lymphomas [22], patients were immunized with the idiotypes of their tumors and monitored for disease free survival. Two randomized trials failed to show statistically significant clinical benefit in the idiotype-immunized arm [23, 24], while a third trial did show such difference [25].

Among solid tumors, a series of randomized trials in patients with colon cancers was performed where patients were immunized post-surgical resection, with whole irradiated autologous tumor cells mixed with BCG, or were not immunized. The last such randomized trial showed statistically significant benefit in the immunized patients with stage II, but not stage III colon cancer [26].

The heat shock protein (HSP)-based vaccine is yet another way to harness the antigenic individuality of each cancer. This approach is based on the demonstration that molecules of HSPs of the hsp70 and hsp90 families are associated non-covalently (1:1 or 2:1) with a broad array of peptides generated in the cells during proteolytic degradation [27]. These peptides consist overwhelmingly of self-peptides, but also contain any non-self peptides generated in the source from which the HSPs are isolated. Such non-self peptides include viral peptides [28] (if HSPs were isolated from virus-infected or transformed cells) or tumor antigenic peptides if the HSPs were isolated from tumor tissues [29, 30]. Thus, the purified HSP preparations are actually HSP-peptide preparations. Upon immunization, the HSP-peptide complexes are taken up by antigen presenting cells of the host through HSP receptors [31, 32], and the peptides are cross-presented by the MHC molecules of the antigen presenting cells, which then engage the T cells, and mediate anti-tumor responses. A Phase 3 trial in patients with renal cell carcinoma in the adjuvant setting, where each patient was immunized with HSP-peptide complexes isolated from his/her own tumor, failed to show statistically significant clinical activity in the overall population, although significant activity was observed in post hoc sub-sets of early and intermediate stage disease [2]. A large randomized trial using this approach is currently underway in patients with glioblastoma multiforme.

There are two ways to look at this history of individually specific vaccination against cancers. At first look, none of these three approaches have succeeded: None is widely used in cancer therapy today. The idiotype vaccine for B cell lymphoma and the whole cell vaccine for colon cancer showed statistically significant clinical activity, but are encumbered by difficulties in vaccine production or regulatory concerns about vaccine quality. The HSP-based vaccine failed to show statistically significant activity except in post hoc sub-sets and, although approved for use in Russia, has not been cleared for use in the US or Europe. All three vaccines are under further improvement. However, if one looks at these three vaccines in the larger universe of all cancer vaccines tested in Phase 3 trials, a somewhat different pattern emerges. With a single exception, all of the vaccines based on the idea of common antigenicity of cancers have failed [1, 33], and the only one that was approved for use in the US [3] is struggling to achieve acceptance, partly because of lack of confidence in its clinical activity. The results of ongoing randomized clinical trials in patients with melanoma and lung cancer vaccinated with the shared, un-mutated vaccine MAGE, as also those of improved versions of the three autologous vaccines discussed here, will bring some clarity to the picture or may muddy it further. Regardless, scientific and clinical data to date provide strong evidence for the existence of an individually distinct antigenic repertoire for each individual cancer, and the feasibility of using this repertoire for successful cancer therapy. This theme is developed further in the next section.

## Harnessing the individually distinct immunome of each individual cancer: the genomic way

What is this antigenic repertoire that is individually distinct for each cancer? What are its components? How is it generated? 20 years ago, I suggested that randomness of passenger mutations in individual tumors generates this repertoire [34]. The argument can be unfolded thus: because the process of DNA replication is not completely accurate, and each cell division in any cell leads to a small number of errors, even after the repair mechanisms have corrected most of the errors. This error rate can range anywhere between one error in a billion to one error in a hundred thousand base pairs replicated per cell division depending upon the cell type and the degree of genomic instability in it [35, 36]. Even at the lowest rate of errors, a tumor will accumulate thousands of mutations by the time it progresses from the first transformed cell to a clinically or radiologically detectable tumor. Simply by statistical probability, a small proportion of these mutations will create new epitopes for some of the MHC I alleles of the tumor. I had suggested that (a) such neo-antigens will be created by the passenger mutations that have nothing to do with the transformed phenotype and that may or may not confer any survival advantage to the tumor, and (b) since these are random mutations, their repertoire for any particular tumor is likely to be unique. This mechanism would explain the unique antigenicity of tumors as observed by earlier workers as discussed above. At the time this mechanism was predicted (1993), high throughput DNA sequencing was still far away, and the possibility that this hypothesis could be experimentally tested did not really exist.

Fast forward to 2008, when high throughput DNA sequencing technologies began to be usable. Using banked samples of breast and colon cancers, and based on partial sequences of tumor transcripts, Segal et al. [37] utilized the algorithms for prediction of HLA binding sequences, and in the first study of this kind, predicted individual breast and colon cancers to have between 7 and 10 new and tumor-specific HLA A201-restricted epitopes!

We made use of probability theory in estimating the number of tumor-specific neo-epitopes in a tumor [38]. Some of the results were entirely expected, but provided the benefit of quantitation, while others were novel. Among the expected results, the analysis showed that the number of potential neo-epitopes (a) varies directly as a function of the mutation rate and (b) increases exponentially with increasing number of cell divisions (i.e., the older a tumor, the more neo-epitopes it has). Further, as expected, it showed that the tumors become more antigenically heterogeneous as they grow. In a novel deduction, the analysis showed that the death rate within a tumor has a profound effect on its immunogenicity. A tumor with a higher death rate will require many more cell divisions to achieve a certain mass as compared to a tumor with a lower death rate. Therefore, a tumor with a higher intrinsic death rate will be more immunogenic. This result places tumor immunogenicity at the intersection of a number of non-immunological characteristics such as tumor vascularization, hypoxia, size and remains to be fully understood or exploited.

Predictions and theoretical considerations aside, the first actual effort at genomics-guided definition of tumor-specific epitopes was published by Sahin and colleagues [39]. Using exome sequencing of a cell line derived from the spontaneous mouse melanoma B16, Castle et al. uncovered tens of neo-epitopes generated by mis-sense mutations and characterized them with respect to their immunogenicity; they observed that a significant proportion of the predicted neo-epitopes was actually immunogenic in vivo. They also showed that immunization with two of such neo-epitopes modulated the course of tumor growth in tumor-bearing and prophylactically treated animals. These findings, important in and of themselves, were particularly interesting because they were made in a poorly immunogenic tumor line.

Schreiber and colleagues used high throughput DNA sequencing to build on their work on immunoediting of cancers [40]; they identified a mutation-generated epitope in a tumor arising in an immunodeficient mouse, and showed that this neo-epitope becomes a tumor-rejection antigen upon transplantation into an immunocompetent mouse, and becomes the subject of immunoediting.

Our laboratory has carried out genomics-guided identification of several chemically induced and spontaneous mouse tumors [41]. Using methods broadly similar to those of Castle et al. [39], but with significant differences, these studies have un-covered hundreds of epitopes in the chemically induced tumors and a much smaller number in the spontaneous tumors and have shown a proportion of them to be immunogenic in vivo.

Collectively, the genomics-driven approach to identification of tumor-specific neo-epitopes has just begun and is beginning to support the postulate [34] that (a) tumors do harbor an individually distinct repertoire and (b) that this repertoire is created by randomness of passenger mutations. While the previous approaches to harnessing this individually specific repertoire [26, 34] were handicapped by the inability to actually identify this repertoire for individual tumors, the new genomics technologies promise to help overcome that critical hurdle.

## Challenges in translation to the human setting

The genomics-driven approach to harnessing the individually distinct repertoire of tumor-specific mutations requires significant enquiry and resolution in mouse models; regardless, it may not be entirely out of place to begin to consider the challenges in translating this approach to the human situation. Rapid and cheap high throughput sequencing of exomes or transcriptomes is not a challenge anymore and most core facilities at academic institutions as well as commercial facilities do this readily. Bio-informatic analysis of such sequences is also becoming more widely accessible through pipelines already generated. However, a number of key challenges remain.

### Selection of candidates of immunization

It is clear that a pipeline of potentially immunogenic epitopes can be generated through analyses in silico [42, 43]. The challenge is to trim this (expected-to-be-quite-long) list into a list that is small enough to be practical and contains epitopes that will be truly tumor protective. (See also the issue of antigenic heterogeneity below.) Not all immunogenic epitopes will be tumor protective, and we cannot reasonably immunize patients with all the putative epitopes identified in silico. A better understanding of this question is perhaps the single most significant challenge in translating genomics into true tumor immunomics.

Another issue greatly worthy of consideration is the possibility that immunization with a mutated epitope may elicit cross-reactive T cell response against the wild-type epitope as well. This raises the specter of at least some degree of autoimmunity, which may or may not be pathological. One may draw some lesson from the fact that immunizations of patients with un-mutated self-epitopes have seldom elicited pathological autoimmunity [1, 3]. Regardless, there is need for caution in this regard, and only further studies in mice and humans shall clarify this issue.

### Technology of immunization

How do we immunize? Do we use a collection of GMP-grade peptides or do we use RNA encoding multiple epitopes [44, 45]? What adjuvants do we use? How much immunogen should be used? What should be the regimen of immunization? These questions do not require a conceptual leap, but they do need considerable examination and experimentation.

### Antigenic heterogeneity

**Fig. 1:**
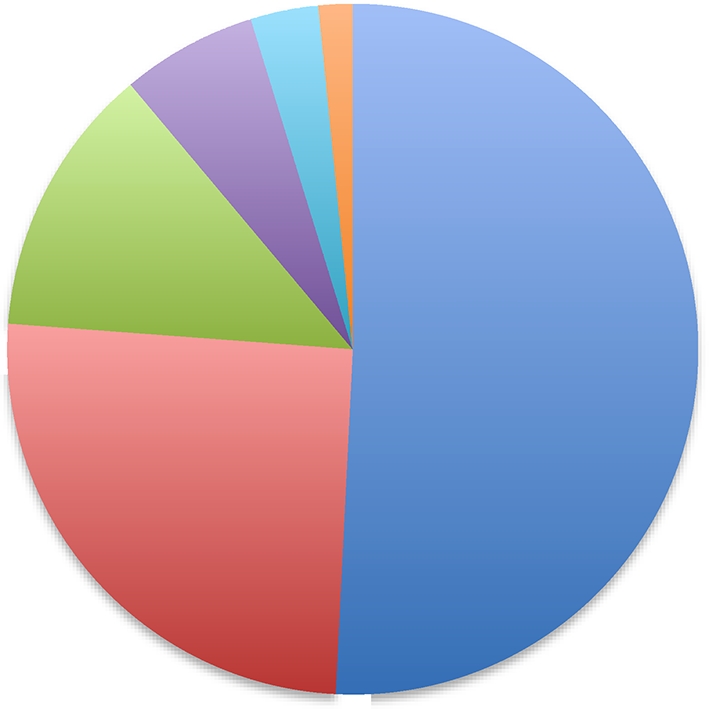
Antigenic heterogeneity in tumor masses. A schematic showing the emergence of random passenger mutations (that are not required for the transformed phenotype and that do not confer any survival advantage or disadvantage and assuming zero tumor cell death) in a growing tumor mass. The mutation that occurs at the first division of the transformed cell is imprinted on 50 % of the population, while mutations occurring in subsequent cell cycles (*red*, *green*, *purple*, *turquoise,* and *orange*, in that order) are presented on increasingly narrower population segments, leading to a tumor with various sub-population of cells expressing different sets of mutations. The figure may appear to suggest (incorrectly) that tumors are actually compartmentalized in this manner: Since newer mutations are as likely to occur in cells that harbor older mutations as in the cells that do not, the tumor mass will actually be a chimera of cells presenting large numbers of overlapping sets of neo-epitopes

This issue is a large one, but arguably less significant than it may appear. The idea that tumor-specific neo-epitopes are generated by random mutations inherently harbors the idea of extensive antigenic heterogeneity: The mutations that occurred earlier in the clonal expansion of a tumor are likely to be imprinted on a larger proportion of tumor cells than those that occur later, assuming that both classes of mutations are neutral with respect to any survival advantage or disadvantage on cells. This scenario creates an image of a highly compartmentalized tumor-cell population, which contains a large number of epitopes presented by narrower and narrower segments of the tumor (Fig. 1). The reality is actually likely to be different from that caricaturized in Fig. 1. The new mutations are as likely to occur in cells that harbor the older mutations as in cells that do not (see [46–49] for stimulating discussion). The net result would be not a compartmentalized population of tumor cells as in Fig. 1, but a tumor mass that is a hopelessly mixed chimera of cells that each contain different sets of overlapping epitopes. The challenge would be to immunize with a large enough cocktail of overlapping epitopes. It should be possible to identify this cocktail for any given tumor (and its metastatic progeny) by the use of a suitable combination of sequencing and bio-informatic methods. This is important work that needs to be done, but can be done. Finally in this regard, it is worth remembering that one does not need to eliminate 100 % of the cells to obtain significant clinical benefit; bystander killing of antigen-negative tumor cells is a robust reality [50, 51].

### Regulatory challenges

There are obvious regulatory challenges in the use of an individual-specific platform of immunotherapy. The reasonable requirements of quality controls for each lot of drug are far more complex in an individualized therapy (where the drug made for each individual patient is a new drug lot) than in a traditional therapy where a single lot caters to a large patient population. However, many if not most of these regulatory challenges have already been addressed to a significant degree since a large number of clinical trials (including randomized multi-center Phase 3 clinical trials) have been conducted previously with individual-specific immunotherapies [2, 26].

Meeting these challenges is our immediate task. To quote the 16th President of the United States, “As our case is new, so we must think anew, and act anew.” [52].
